# Spotted Fever Group Rickettsiae in Ticks and Small Mammals from Grassland and Forest Habitats in Central Germany

**DOI:** 10.3390/pathogens12070933

**Published:** 2023-07-12

**Authors:** Charlotte Arz, Nina Król, Christian Imholt, Kathrin Jeske, Zaida Rentería-Solís, Rainer G. Ulrich, Jens Jacob, Martin Pfeffer, Anna Obiegala

**Affiliations:** 1 Institute of Animal Hygiene and Veterinary Public Health, Faculty of Veterinary Medicine, University of Leipzig, An den Tierkliniken 1, 04103 Leipzig, Germany; c.arz@studserv.uni-leipzig.de (C.A.); nina.krol@vetmed.uni-leipzig.de (N.K.); pfeffer@vetmed.uni-leipzig.de (M.P.); 2 Institute for Epidemiology and Pathogen Diagnostics, Julius Kühn-Institute, Toppheideweg 88, 48161 Münster, Germany; christian.imholt@julius-kuehn.de (C.I.); jens.jacob@julius-kuehn.de (J.J.); 3 Institute of Novel and Emerging Infectious Diseases, Friedrich-Loeffler-Institut, Südufer 10, 17493 Greifswald, Germany; kathrin.jeske@gmx.de (K.J.); rainer.ulrich@fli.de (R.G.U.); 4 Institute for Parasitology, Centre for Infectious Diseases, Faculty of Veterinary Medicine, University of Leipzig, An den Tierkliniken 35, 04103 Leipzig, Germany; zaida_melina.renteria_solis@uni-leipzig.de

**Keywords:** *Ixodes*, *Dermacentor*, *Apodemus*, *Sorex*, *Microtus*, *Clethrionomys*, season, life stage, ecotone, *Borrelia*

## Abstract

Rickettsiae of the spotted fever group (SFG) are zoonotic tick-borne pathogens. Small mammals are important hosts for the immature life stages of two of the most common tick species in Europe, *Ixodes ricinus* and *Dermacentor reticulatus*. These hosts and vectors can be found in diverse habitats with different vegetation types like grasslands and forests. To investigate the influence of environmental and individual factors on *Rickettsia* prevalence, this study aimed to analyse the prevalence of SFG rickettsiae in ticks and small mammals in different small-scale habitats in central Germany for the first time. Small mammals of ten species and ticks of two species were collected from grasslands and forests in the Hainich-Dün region, central Germany. After species identification, DNA samples from 1098 ticks and ear snips of 1167 small mammals were screened for *Rickettsia* DNA by qPCR targeting the *gltA* gene. Positive samples were retested by conventional PCR targeting the *ompB* gene and sequencing. *Rickettsia* DNA was detected in eight out of ten small mammal species. Small mammal hosts from forests (14.0%) were significantly more often infected than those from grasslands (4.4%) (*p* < 0.001). The highest prevalence was found in the mostly forest-inhabiting genus *Apodemus* (14.8%) and the lowest in *Microtus* (6.6%), which inhabits grasslands. The prevalence was higher in *D. reticulatus* (46.3%) than in the *I. ricinus* complex (8.6%). Adult ticks were more often infected than nymphs (*p* = 0.0199). All sequenced rickettsiae in *I. ricinus* complex ticks were *R. helvetica*, and the ones in *D. reticulatus* were *R. raoultii*. Unlike adults, questing nymphs have had only one blood meal, which explains the higher prevalence in *I. ricinus* adults. Interestingly, habitat type did influence infection probability in small mammals, but did not in ticks. A possible explanation may be the high prevalence in *Apodemus flavicollis* and *A. sylvaticus* which were more abundant in the forest.

## 1. Introduction

Hard ticks are haematophagous arthropods that can be found in diverse habitats with different kinds of vegetation. While feeding, they may serve as vectors for multiple tick-borne pathogens (TBPs) like protozoan parasites, bacteria, and viruses. The most common tick species in central Europe is the castor bean tick *Ixodes ricinus* [1]. It can be found in various environments, from forests over grasslands to urban areas [2]. As three-host non-nidicolous ectoparasites, immature *I. ricinus* ticks (larvae and nymphs) feed on small and medium-sized mammals, birds, and reptiles, while adult ticks rather feed on larger mammals such as roe deer (*Capreolus capreolus*) and wild boar (*Sus scrofa*) [3]. The geographical movement of the hosts is essential for the distribution of ticks and their carried pathogens [4]. The ornate dog tick, *Dermacentor reticulatus*, is also a commonly found tick species in Germany and is linked to many TBPs [1]. Its immature life stages are nidicolous and feed on small mammals, while non-nidicolous adults usually feed on larger mammals [5].

In general, small mammals act as reservoirs for many vector-borne pathogens such as tick-borne alpha proteobacteria belonging to the order Rickettsiales or spirochaetes from genus *Borrelia* [6]. Thus, the composition of the natural habitat plays an important role in the occurrence and diversity of small mammal species [7], and in turn, may have an impact on the occurrence and density of hard ticks and their TBPs [8].

*Rickettsia* spp. are obligate intracellular bacteria and can be divided into four groups: the spotted fever group (SFG), the typhus group (TG), the *R. canadensis* group, and the *R. bellii* group [9]. Rickettsiae have been detected on all continents except Antarctica [9] and are mostly transmitted to mammals, including humans, reptiles, and birds, by haematophagous arthropods like fleas, lice, ticks, and other mites through blood meals or contaminated faeces [10]. Forming the most numerous group within the genus, the SFG is the most widespread in Europe and almost exclusively tick-borne, with only two exceptions: *R. felis* and *R. acari* [11]. *Rickettsia* spp. transmission between ticks has been confirmed for transstadial, sexual, and transovarial pathways, and also, but rather rarely, through co-feeding of *I. ricinus* ticks [12,13,14,15]. Even though different transmission paths exist within a population of ticks, reservoir hosts seem to play an important role in maintaining the life cycle and distribution of the bacteria as well. DNA of *R. helvetica* was detected in roe deer and wild boar, and therefore these mammal species are handled as potential reservoir hosts [16]; however, small mammals are assumed to be the main reservoir hosts [17].

Both *R. helvetica* and *R. monacensis* are part of the SFG and are associated with *I. ricinus*. *Rickettsia helvetica* is the most commonly found *Rickettsia* species in Germany [18,19,20,21] and is considered pathogenic due to multiple reports of clinical symptoms like fever, headaches, and myalgia in connection with *R. helvetica* infections in humans [9,22]. *Rickettsia monacensis* may rarely cause a rickettsiosis [23].

*Rickettsia raoultii,* also part of the SFG, is usually found in *D. reticulatus* and *D. marginatus* and has been detected in small mammals as well [20]. This species is associated with a syndrome called “SENLAT”, which stands for scalp eschars and neck lymphadenopathy after tick bite [1,9,24]. The syndrome has been diagnosed in various countries across Europe, including Germany; however, not in all cases *R. raoultii* has been confirmed as the causative agent [24,25,26]. Likewise, *R. slovaca* may cause these symptoms and is transmitted by the same vectors [27]. Studies on co-infections of *Rickettsia* spp. and *Borrelia* spp.—causing the most common tick-borne disease in Europe, Lyme disease—in ticks and especially in small mammals are rare [28]. *Rickettsia* spp. prevalence in ticks has been analysed earlier regarding different factors like season, tick life stage, and multiple landscape factors [29,30,31]. However, observations of *Rickettsia* prevalence in ticks, including other demographic factors such as habitat and small mammal species composition, plus *Rickettsia* prevalence in small mammals in the context of habitat structure are scarce in Europe. To fill these knowledge gaps, in this study, we (1) analysed questing ticks and small mammals at differently structured study sites around the Hainich-Dün National Park in central Germany, (2) investigated their SFG *Rickettsia* prevalence, (3) identified the *Rickettsia* species, (4) analysed co-infection rates of *Rickettsia* spp. and *Borrelia* spp. in ticks and small mammals, and (5) investigated the influence of environmental and individual host and vector factors like habitat, species of small mammals and ticks, ticks’ life stage, and season on *Rickettsia* prevalence.

## 2. Materials and Methods

### 2.1. Study Sites, Sample Collection, and DNA Extraction

#### 2.1.1. Study Sites

In total, 21 study sites surrounding the Hainich-Dün National Park in Thuringia, central Germany were examined. The area is one of the largest continuous deciduous forests in Germany, with European beech (*Fagus sylvatica*) being the dominant tree species. The region is characterised by forest and agricultural areas which are cultivated to different extents. While some forests and grasslands are extensively managed, the forests of the National Park itself are protected (https://www.biodiversity-exploratories.de/en/regions/hainich-duen/, accessed on 25 April 2023). A more detailed description of the study area was given in Król et al. [32].

#### 2.1.2. Tick Collection

**Figure A1:**
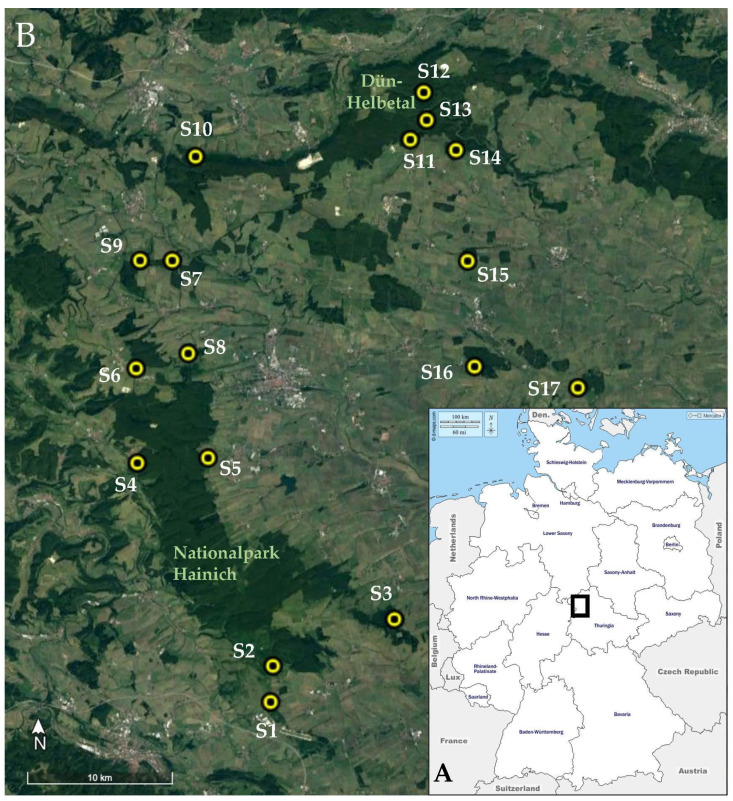
(**A**) Location of study sites in Germany (inset). (**B**) Study sites with *Rickettsia* DNA positive samples. S = Study site. The image was created using Google Earth Pro, Map: Google Earth ©2023 Google, Image Landsat/Copernicus ©2023 GeoBasis-DE/BKG.

A total of 1115 questing ticks were collected for a previous study by flagging 100 m^2^ simultaneously with small mammal trapping at 17 of the 21 sites, once per season (spring, summer, and autumn) in 2018 and 2019 [32] (Figure A1). The study sites for ticks were composed of one plot in the forest and one in the bordering grassland–forest ecotone, as described before [32]. Collected ticks were specified for sex, life stage, and species under a light microscope (Motic^®^ SMZ–171, Moticeurope, S.L.U., Barcelona, Spain) according to taxonomic keys [33,34]. DNA was extracted using a QIAmp DNA Mini Kit (Qiagen, Hilden, Germany) according to the manufacturer’s instructions for DNA isolation. Extracted DNA of 1094 ticks was available from the study of Król et al., where further details of tick handling, taxonomic identification, and DNA extraction procedure are described [32]. In addition to the given dataset, four ticks (three nymphs and one female of *I. ricinus*) that were all collected in spring 2019 were processed after the same protocol. Seventeen individuals (four *I. ricinus* nymphs and thirteen *D. reticulatus* adults) had to be excluded from further molecular biological analyses due to insufficient material conservation. Data on *I. ricinus* and *I. inopinatus* (presumably 16 individuals) were merged under the terminus “*I. ricinus* complex” [35].

In total, 1115 ticks belonging to 2 species were collected (Table A1). The most prevalent species was the *I. ricinus* complex (91.7%), followed by *D. reticulatus* (8.3%). The most frequently found life stages were the nymphs of the *I. ricinus* complex (74.9%). Most ticks were flagged in spring (74.1%) in comparison to summer (18.3%) and autumn (7.6%). In ecotones less ticks (35.2%) were flagged than in forests (64.8%). After excluding the above-mentioned ticks, 1018 ticks of the *I. ricinus* complex and 80 individuals of *D. reticulatus* were further processed (Table 1).

#### 2.1.3. Small Mammal Collection

Extracted DNA derived from small mammal skin samples was available from a former study [32]. The samples were taken from about 0.5 cm × 0.5 cm ear tissue. DNA extraction was performed using the same methodology as for the ticks. Small mammals were trapped at 21 study sites, each in a paired system of a forest plot and an adjacent grassland plot per site [32] (Figure A1).

Snap trapping of small mammals took place in spring, summer, and autumn in 2017–2019. Trapping procedures, dissections, and further processing of samples have been published elsewhere [36,37]. For the current study, 1167 DNA samples that were randomly picked from 1945 individuals were available from summer 2017 and spring and summer 2018 and 2019 [32]. Extracted DNA belonged to 10 small mammal species of the families Soricidae, Cricetidae, and Muridae. The species collected most often was the common vole (*Microtus arvalis*) (*n* = 407; 34.9%), followed by the bank vole (*Clethrionomys glareolus*) (*n* = 278; 23.8%), the yellow-necked mouse (*Apodemus flavicollis*) (*n* = 240; 20.6%), the long-tailed field or wood mouse (*Apodemus sylvaticus*) (*n* = 108; 9.3%), and the striped field mouse (*Apodemus agrarius*) (*n* = 90; 7.7%). Individuals belonging to the following species were rather occasionally captured (all ≤ 20 individuals): the common shrew (*Sorex araneus*), the field vole (*Microtus agrestis*), the European water vole (*Arvicola amphibius*), the Eurasian pygmy shrew (*Sorex minutus*), and the greater white-toothed shrew (*Crocidura russula)* [32] (Table 2).

### 2.2. Real-Time PCR, Conventional PCR and Sequencing

Quantitative real-time PCR (qPCR) was used to screen all tick and small mammal samples for a 70 base pair (bp)-sized region of the citrate synthase gene (*gltA*) of *Rickettsia* spp. The mix was prepared as described previously [38] using the LightCycler^®^ FastStart DNA Master HybProbe (Roche Diagnostics GmbH, Mannheim, Germany). The cycling protocol for the Thermocycler (Stratagene Mx3000P, Agilent, Santa Clara, CA, USA) included 95 °C for 10 min followed by 45 cycles of denaturation at 95 °C for 25 s, annealing at 55 °C for 30 s, and extension at 72 °C for 30 s.

Tick samples tested with a cycle threshold (ct) < 36 and small mammal samples tested with a ct < 35 were subsequently examined by conventional PCR targeting a fragment of the gene encoding for the outer membrane protein B (*ompB*, 811bp) of the SFG rickettsiae. A previously described protocol [39] was followed with the primers “120–2788” and “120–3599” with one adjustment of the initial denaturation temperature of 94 °C.

For visualisation of the PCR products, gel electrophoresis was performed—8 µL of the samples were mixed with 2 µL of loading dye (TriTrack DNA Loading Dye (6×), Thermo Scientific™, Waltham, MA, USA) and separated on a 1.5% agarose gel.

PCR products were prepared for sequencing using the NucleoSpin^®^ Gel and PCR Clean-up kit (Macherey-Nagel GmbH & Co. KG, Düren, Germany) according to the protocol recommended by the manufacturer. Sequencing was performed commercially by Eurofins Genomics Germany GmbH (Ebersberg, Germany). After editing and aligning the sequences with Bionumerics (Applied Maths NV, Sint-Martens-Latem, Belgium), a comparison was conducted to sequences present in GenBank on the Basic Local Alignment Tool (BLAST; https://blast.ncbi.nlm.nih.gov/Blast.cgi, accessed on 9 March 2023). Two obtained sequences from small mammals and 49 obtained sequences from ticks from 2019 were submitted to GenBank under the accession numbers OQ694692–OQ694742.

### 2.3. Statistical Analysis

For *Rickettsia* spp. prevalence of ticks and small mammals, a confidence interval (95% CI) was formulated using the Clopper and Pearson method with Graph Pad software (GraphPad Software, San Diego, CA, USA). For small mammals, a generalised linear mixed model (GLMM) with binomial error distribution was computed with R software (version 4.1.2. for Windows; RStudio, Boston, MA, USA) and the *lme4* package. It was used to investigate the dependence of individual *Rickettsia* infection status (dependent binary variable; *Rickettsia* spp. positive = 1; *Rickettsia* spp. negative = 0) in relation to season (independent binary variable: summer vs. spring), habitat (independent binary variable: forest vs. grassland), and small mammal species (independent categorical variable) [40]. Because of low numbers of trapped individuals, the following five species were excluded from GLMM analysis: *M. agrestis*, *A. amphibius*, *S. araneus*, *S. minutus*, and *C. russula*.

In addition, GLMMs were generated for *A. flavicollis*, *Cl. glareolus,* and *M. arvalis* separately. Independent variables were sex of the individual (independent binary variable: female vs. male), habitat (see above), and season (see above).

For ticks, a slightly different model was calculated. The dependent variable was the binominal “presence of *Rickettsia*”. The independent variables were (i) developmental stage (binary: adult vs. nymph), (ii) habitat (binary: forest vs. ecotone), (iii) season (three stages: autumn, spring, and summer), and (iv) tick species (binary: *I. ricinus* complex vs. *D. reticulatus*). The first three independent variables were used for a model with exclusively *I. ricinus* complex ticks. Due to the low number of individuals collected, the model was not carried out for *D. reticulatus* ticks.

The interaction term for the GLMM included three and four independent variables with at least two levels each for small mammals and *I. ricinus* complex ticks, and ticks, respectively. To state potential differences of all variables separately, marginal means accessing the *emmeans* package within R and a post hoc test were computed. The significance threshold for *p* was set at ≤0.05.

### 2.4. Co-Infections of Borrelia spp. and Rickettsia spp. in Ticks and Small Mammals

Results of detection of *Borrelia* spp. DNA in small mammals (1167 individuals) and tick samples (1094 individuals) that were also investigated in this study have been published in an earlier study [32]. In the current study, co-infections of *Borrelia* spp. (previous study) with *Rickettsia* spp. (current study) were analysed. To investigate the degree of co-occurrence, the approach proposed by Malini et al. was applied [41]. Here, the R package *CooccurrenceAffinity* was applied to calculate a maximum likely estimator to evaluate whether or not the pairing of *Rickettsia* spp. and *Borrelia* spp. was more or less likely than the expected prevalence [42]. Seasonal analyses were made for ticks and small mammals separately, and all trapped individuals regardless of species were taken into account.

## 3. Results

### 3.1. Rickettsia spp. Detection in Ticks

Positively tested individuals were from both the *I. ricinus* complex and *D. reticulatus* (Table 1). Samples of *D. reticulatus* (*n* = 37; 46.3%; 95% CI: 35.0–57.8) were significantly more often infected than *I. ricinus* complex samples (*n* = 88; 8.6%; 95% CI: 7.0–10.5) (*p* < 0.001) (Table A2). Per tick species, females had the highest prevalence (21.1%; 95% CI: 12.5–32.0 in *I. ricinus* complex and 52.9%; 95% CI: 38.5–67.1 in *D. reticulatus*), followed by males (8.1%; 95% CI: 3.8–14.8 in *I. ricinus* complex and 34.5%; 95% CI: 17.9–54.3 in *D. reticulatus*) and nymphs (7.6%; 95% CI: 5.9–9.6). Consequently, adults were significantly more often carriers of *Rickettsia* DNA than immatures, overall (*p* = 0.02) and regarding only the *I. ricinus* complex (*p* = 0.005). The prevalence in the forest (8.8%; 63/720; 95% CI: 6.79–11.06) was similar to the ecotone (16.4%; 62/378; 95% CI: 12.8–20.5) (*p* = 0.711). In spring the prevalence was the highest with 12.5% (103/826; 95% CI: 10.3–14.9), followed by summer with 9.4% (19/203; 95% CI: 5.7–14.2) and autumn with 4.3% (3/69; 95% CI: 0.9–12.2). Ticks in spring and summer were both more often infected than in autumn (spring: *p* < 0.001; summer: *p* < 0.001) (Table A2).

Out of all qPCR-tested ticks, 125 yielded a ct-value < 36, 87 were positive in conventional PCR, and 86 could be sequenced and identified as *R. helvetica* (86.0%; *n* = 74, all *I. ricinus* complex) and *R. raoultii* (14.0%; *n* = 12, all *D. reticulatus*) (Table 3). Most *Rickettsia*-positive *I. ricinus* complex samples showed 99.5–100% identity with the same accession number as the small mammal samples (see below) [43]. One sample from *I. ricinus* was 100% identical to *R. helvetica* isolate Komi (GenBank accession number: KP866151), sequenced from *Ixodes persulcatus* ticks from the Komi Republic in Russia [44]. Most rickettsiae of *D. reticulatus* ticks showed 99.9–100% identity with *R. raoultii* Xinjiang-CP13RP (GenBank accession number: MG811717) from China. One *D. reticulatus* tick-derived *Rickettsia* sequence was 99.9% identical to the *R. raoultii* clone ALSK081 (GenBank accession number KU723537) from Xinjiang, China, and two were 99.9% and 100% identical to the *R. raoultii* isolate Xinjiang-JMN (GenBank accession number: MF002526) from China.

### 3.2. Rickettsia spp. Detection in Small Mammals

*Rickettsia* spp. DNA was detected in 8 out of 10 small mammal species (*A. flavicollis*, *n* = 37; *M. arvalis*, *n* = 26; *A. sylvaticus*, *n* = 20; *Cl. glareolus*, *n* = 19; *A. agrarius*, *n* = 8; *S. araneus*, *n* = 2; and *M. agrestis*, *n* = 2; *S. minutus*, *n* = 1) (Table 2).

In summer (10.2%; *n* = 102; 95% CI: 8.4–12.3) prevalence was similar to spring (7.7%; *n* = 13; 95% CI: 4.2–12.9) (*p* = 0.106). Individuals from forests (*n* = 93; 14.0%; 95% CI: 11.5–16.9) were more often infected than individuals from grassland (*n* = 22; 4.4%; 95% CI: 2.7–6.5) (*p* < 0.001). *Apodemus sylvaticus* had the highest prevalence of 18.5% (*n* = 20; 95% CI: 11.7–27.1).

The GLMM with the factors of season and habitat (Table A3) revealed statistical significance for the lower probability of infection of an individual of *Cl. glareolus* compared to *A. agrarius* (*p* = 0.034). No statistical significance was observed when only the two species were compared and no other factors were taken into account (*p* = 0.206). *Clethrionomys glareolus* was less often infected with *Rickettsia* spp. than both *A. flavicollis* (*p* = 0.007) and *A. sylvaticus* (*p* < 0.001).

In the GLMMs computed individually for the three most abundant species, sex and season did not influence the prevalence in *M. arvalis*, *Cl. glareolus* and *A. flavicollis* (Table A3). Out of these three species, habitat mattered only for *M. arvalis*. The prevalence was higher in forests, but 92.6% of the animals of that species were trapped in grasslands (*p* < 0.001) (Table 2).

Although excluded from the analysis with GLMM, this is the first detection of *Rickettsia* spp. DNA in *S. minutus* in Germany to our knowledge.

Of the 115 individuals that were tested positive in qPCR, 24 yielded a ct-value <35. Only two of them (both *A. flavicollis*) yielded a result in the *ompB* gene. Both amplicons could be sequenced and were 100% identical to *R. helvetica* strain AS819 (deposited in GenBank: MF163037), which was isolated from an *I. ricinus* tick [43] (Table 3).

### 3.3. Co-Infection with Rickettsia spp. and Borrelia spp. in Ticks and Small Mammals

In nine ticks, both *Rickettsia* DNA and *Borrelia* DNA were detected (9/1094; 0.8%). All sample derived from the *I. ricinus* complex (9/1014; 0.9%). The only *Rickettsia* spp. detected was *R. helvetica*. *Borrelia valaisiana* and *Borrelia afzelii* were detected in two samples each. In five tick samples, Borrelia species could not be determined (Table A4). In small mammals, DNA of both bacteria were also found in nine samples (9/1167; 0.8%). Co-infections were detected in *M. arvalis* (*n =* 3; 0.7%), *Cl. glareolus* (*n =* 3; 1.1%), *S. araneus* (*n =* 2; 10.0%), and *A. flavicollis* (*n =* 1; 0.4%). No PCR products of small mammal samples could be analysed to the species level of *Rickettsia* spp. and *Borrelia* spp. (Table A5) [32].

Co-occurrence analysis revealed that *Rickettsia* spp. prevalence in ticks from spring had a slightly negative tendency with *Borrelia* spp. occurrence (Alpha MLE: −0.72, Blaker CI (−1.60–0.004), *p =* 0.06). In all other scenarios, no co-occurrence trends were observed (Table A6).

## 4. Discussion

Rickettsiae of the SFG are pathogens of public health concern. Studies, including analyses of *Rickettsia* prevalence in both ticks and small mammal hosts regarding various aspects, like particular habitats and the possible influence on each other, are largely overlooked. This study presents, for the first time, data on *Rickettsia* species and their prevalence in ticks and small mammals in central Germany with different habitat features.

In the current study, the most prevalent tick species was *Ixodes ricinus*, which is the most abundant tick species in Germany [45]. *Ixodes ricinus* ticks in central Europe have a bimodal activity pattern peaking in warm and humid months, with a larger peak in spring and a smaller peak in autumn. In the Mediterranean area, a unimodal activity pattern with one big peak in spring is common, which is now also more often observed in central Europe, as shifts in temperature and precipitation result in a decline in the impact of microclimatic conditions like sufficient humidity [46,47]. In our study, we also did not find an activity peak in autumn, but observed a unimodal activity pattern with a peak in spring for nymphs and adults. The ratio of *I. ricinus* complex adult ticks collected in ecotones and forests was similar, fitting their described primary habitat of forests and shrubbery [48]. In all three considered seasons, *I. ricinus* complex nymphs were more often collected in the forest compared to the ecotone. One previous study from southern Germany showed a positive effect of humidity on the occurrence of nymphs [46]. In the forest areas investigated, a base layer with higher humidity serves as protection from desiccation during hotter months, explaining why more ticks were collected in the forest sites compared to the ecotone [2].

Less than a tenth of the collected ticks belonged to the species *Dermacentor reticulatus* [45]. Larvae and nymphs of *D. reticulatus* commonly show nidicolous behaviour and live in burrows and nests of small mammals [33]. This is why only adult individuals were collected in the current study. *Dermacentor reticulatus* ticks show a quiescence phase over summer and two activity peaks, one in spring and one in autumn [24,49], which we observed in our study. Most *D. reticulatus* ticks (93.6%) were flagged in the ecotone which is in concordance with the described natural habitat of bushy pastures, meadows, and open forests [48,49].

As expected, most *Clethrionomys glareolus* (97.1%) and *Apodemus flavicollis* (91.7%) were trapped in the forest. Opposite to that, *Microtus arvalis* was mostly trapped in grasslands (92.6%). This distribution matches the preference of *Cl. glareolus* and *A. flavicollis* for forests and *M. arvalis* for grasslands [50,51]. *Apodemus sylvaticus* was trapped with a proportion of 74.1% in forests, which reflects their well-known common habitat shift between forests and grasslands [7,52]. Proportions of trapped *A. agrarius*, *Sorex araneus*, and *M. agrestis* were balanced in forest and grassland. These three species do not show such a strong link to either one of the two considered habitats [7,51,53].

Small mammals serve as reservoirs for many zoonotic pathogens including rickettsiae of the SFG group of diverse pathogenicity. In Germany, most known severe human cases were, however, not autochthonous [54]. The two most common species in Germany, *Rickettsia helvetica* and *R. raoultii,* are nowadays known to cause clinically unspecific symptoms such as eschars in humans [24,55].

In central Europe, DNA of *R. helvetica* and *R. raoultii* has been detected in arthropods such as fleas and various tick species like *I. ricinus*, *D. reticulatus*, and *D. marginatus*. Animals in which rickettsial DNA has been detected include rodents, racoons (*Procyon lotor*), roe deer, wild boars, and lizards [16,20,27,56,57].

To our knowledge, this is the first detection of *Rickettsia* spp. DNA in *S. minutus* from Germany. *Rickettsia* spp. DNA could not be recorded in a former study from Germany, including 72 individuals of *S. minutus*, of which 16 likewise originated from the state of Thuringia [58]. A study from Norway has shown a lower infestation rate of *S. minutus* with ticks compared to investigated individuals from the genus *Apodemus* [59], which has also been displayed for shrews in a study from France [52]. This, in turn, might lead to a lower risk of infection with TBPs. As in our study, *Rickettsia* spp. DNA has been detected in *S. araneus* in other studies before [58,60].

Interestingly, individuals in forests were more often infected than those in grasslands. Among the investigated genera, *Apodemus* had the highest prevalence. As described above, the two most abundant species of the genus *Apodemus*, namely, *A. flavicollis* and *A. sylvaticus*, were mostly trapped in the forest. *Apodemus* spp. showing a higher prevalence than other small mammals has been described before. The prevalence in *A. sylvaticus* (18.5%), *A. flavicollis* (15.4%), and *A. agrarius* (8.9%) falls in line with previous findings from Germany with prevalence ranges of 0–16.2% [58,61], 13.0–23.4% [58,61,62], and 0–9% [58,62], respectively. A few studies on *A. flavicollis* from several European countries showed prevalence rates of 0% in whole blood, 1.7% in organ samples, 5.7% in whole blood, and 29.4% in spleens in Poland [63], Croatia [64], Slovakia [65], and Lithuania [60], respectively. One study from Italy found a prevalence of 6% in ear pinna samples without distinguishing between *Apodemus* species [66].

Difficulties in sequencing of *Rickettsia* spp. DNA in material of small mammal origin due to poor sensitivity of conventional PCR has been described before [58]. In our investigations, only two out of one hundred and fifteen small mammal samples positive in qPCR (24 of which had a ct < 35) could be sequenced. Both were from *A*. *flavicollis* samples and showed the highest similarity to *R. helvetica* strain AS819, which has been isolated from *I. ricinus* ticks before. *Rickettsia helvetica* has been found in *A. flavicollis* in several studies from Germany before [20,58]. One study from the Netherlands found *R. helvetica* in small rodents [16]. *Rickettsia raoultii*, on the other hand, seems to be rather rare in small mammal samples [58]. One study found *R. raoultii* in *Cl. glareolus* which were all infested with *D. reticulatus* [62]. A larger number of sequencing results in our study could have added more certainty as to whether small mammals, in particular *Apodemus* spp., take a less essential part in the life cycle of *R. raoultii*. One study from southern Germany investigated *Dermacentor* ticks and mainly voles without detecting any *Rickettsia* spp. DNA in rodent samples [67]. Two studies from China examined other mammals as potential reservoirs of *R. raoultii* and detected *R. raoultii* in horses (*Equus ferus*) and red foxes (*Vulpes vulpes*) [68,69]. *Rickettsia helvetica* in roe deer and wild boar and *R. slovaca* in wild boar were found in studies from the Netherlands [16] and Algeria [70], respectively.

*Rickettsia* spp. prevalence in tick species differed from 8.6% in the *I. ricinus* complex and 46.3% in *D. reticulatus*. *Dermacentor reticulatus* often shows a higher infection rate with *Rickettsia* than *I. ricinus* [71]. For example, a study from northeast Germany noted a prevalence of 64.0% in *D. reticulatus* [49]. The determined prevalence in the *I. ricinus* complex in our study conforms to prevalences described in other studies on *I. ricinus* from Germany [21,72,73]. However, the methods and life stages of ticks differed among the published studies; therefore, direct comparison of prevalence has to be regarded with caution. Adult ticks showed a statistically higher infection risk than nymphs, which have had only one blood meal as larvae. Nevertheless, it has to be noted that we only analysed nymphs from the *I. ricinus* complex and no immature stages from *D. reticulatus*. In a study also analysing feeding immatures, it has been proposed that transovarial transmission of *Rickettsia* spp. in ticks plays a more important role in *D. reticulatus* than in *I. ricinus* [20]. Success of transovarial and transstadial transmission of *R. raoultii* in *D. reticulatus* have been presented to be 90.0% and 98.0%, respectively [74]. This supports the observation in our study that *D. reticulatus* has a higher prevalence than *I. ricinus.* Also, their preference for voles as hosts, which had a comparably low prevalence in our study, might reinforce this approach.

Regarding only *I. ricinus* ticks, the infection risk was higher in adults than in nymphs. This has been observed in most but not all studies examining exclusively *I. ricinus* [31,72,75]. In spring and summer, ticks were significantly more often infected than in autumn. In the statistical analysis regarding solely *I. ricinus* ticks, no seasonal influence could be noted. In other European studies, in which only *I. ricinus* ticks were considered, the seasonal influence differed. Overall, no pattern can be noted as in some studies from Germany ticks collected in summer and autumn had higher infection rate than in spring [19,76], and in another study ticks collected in summer had a higher infection rate than in autumn and spring [72]. In opposite to that stands the finding from a study from Denmark in which ticks flagged in spring were significantly more often infected than ticks from summer and autumn [77]. In one study analysing only *D. reticulatus* from northeast Germany, no seasonal influence on prevalence was recognised [49].

Habitat type did not influence the prevalence of *Rickettsia* in ticks in our study. Other studies in Europe also did not find any statistical effect of different landscapes on *Rickettsia* prevalence in *I. ricinus* ticks [29,30,31]. It seems that microclimatic factors may play a more important role for *Rickettsia* abundance in ticks than the habitat itself. A study design of field and experimental studies taking the microclimate into account could be useful to identify additional driving parameters for tick infection.

Numbers of co-infections with both *Borrelia* spp. and *Rickettsia* spp. in small mammal species were low, except in *S. araneus* (10%). The co-infection rates in ticks with these two bacteria were low in our study but have been shown to correlate positively before [78]. Another study with a higher prevalence of both bacteria than in our study did not show this positive association [79]. In our study, the only observed effect was a slight tendency of a negative association of *Rickettsia* spp. infection with *Borrelia* spp. occurrence in ticks in spring. Studies investigating co-infections in small mammals are scarce. As *S. araneus* was the only small mammal species with a high co-infection rate in our study and was rarely regarded in previous studies, further research focusing on this correlation should be considered.

In our study, 68.8% of the tick samples that were positive in qPCR could be amplified by conventional PCR, sequenced and identified as either *R. helvetica* or *R. raoultii*. *Rickettsia helvetica* is the most abundant *Rickettsia* species in Germany, as in our study. Interestingly, it was the only *I. ricinus* associated *Rickettsia* species that was determined. Even though *R. monacensis* detection is not that rare in Germany, it seems to occur more often in southern Germany [31,72,73,76]. The *R. helvetica* sequences from our study were identical to those previously found in *I. persulcatus* from Russia and in *I. ricinus* from Germany. *Rickettsia raoultii* sequences found here had been detected in China earlier. Compared to other tick species, *R. raoultii* has a strong link to *D. reticulatus* [9] and was also the only *Rickettsia* species we could detect in *D. reticulatus* in our study. As *I. ricinus* and *D. reticulatus* are the two most common tick species in central Europe, their monitoring is one important tool to gain knowledge about risk factors for human and animal infections.

## 5. Conclusions

The prevalence of *Rickettsia* spp. in small mammals and ticks determined in this study falls in line with previous studies from Germany. Adult ticks had a statistically higher infection risk than nymphs. *Dermacentor reticulatus* showed a significantly higher prevalence than *I. ricinus* ticks. In our study, we found *R. raoultii* and *R. helvetica*, which are both associated with cases of human illness. Interestingly, this study showed no influence of habitat type on the prevalence of *Rickettsia* in ticks but in small mammals, which were significantly more often infected in forests than in grasslands. A possible explanation may be the high prevalence in small mammals of the genus *Apodemus*, which are more abundant in forests. Nevertheless, habitat type should always be considered in a one health perspective, as it has a massive impact on the abundance of potential pathogen reservoirs.

## Figures and Tables

**Table 1:** *Rickettsia* spp. prevalence in ticks tested per habitat.

Tick Species	No. of Individuals in Ecotone Positive/Total (%)	No. of Individuals in Forest Positive/Total (%)	Total No. of Positive Individuals/Total (%)
*Ixodes ricinus* complex ^1^	28/304 (9.2)	60/714 (8.4)	88/1018 (8.6)
*Dermacentor reticulatus*	34/74 (45.3)	3/6 (50.0)	37/80 (46.3)
**Total**	**62/378 (16.4)**	**63/720 (8.8)**	**125/1098 (11.4)**

No.: number. ^1^ *Ixodes ricinus* and *Ixodes inopinatus*.

**Table 2:** *Rickettsia* prevalence in small mammal individuals per species, per habitat, and in total.

Small Mammal Species	No. of Individuals in Grassland Positive/Total (%)	No. of Individuals in Forest Positive/Total (%)	Total No. of Positive Individuals/Total (%)
*Microtus arvalis*	19/377 (5.0)	7/30 (23.3)	26/407 (6.4)
*Clethrionomys glareolus*	1/8 (12.5)	18/270 (6.7)	19/278 (6.8)
*Apodemus flavicollis*	0/20 (0)	37/220 (16.8)	37/240 (15.4)
*Apodemus sylvaticus*	1/28 (3.6)	19/80 (23.8)	20/108 (18.5)
*Apodemus agrarius*	1/46 (2.2)	7/44 (15.9)	8/90 (8.9)
*Sorex araneus*	0/11 (0)	2/9 (22.2)	2/20 (10.0)
*Microtus agrestis*	0/8 (0)	2/7 (28.6)	2/15 (13.3)
*Arvicola amphibius*	0/3 (0)	0/1 (0)	0/4 (0)
*Sorex minutus*	0/2 (0)	1/2 (50.0)	1/4 (25.0)
*Crocidura russula*	0/1 (0)	0	0/1 (0)
**Total**	**22/504 (4.4)**	**93/663 (14.0)**	**115/1167 (9.9)**

No.: Number.

**Table 3:** Sequencing results of PCR products of the *ompB* gene in ticks and small mammal samples.

Habitat	Host Species	Sex /Life Stage	*Rickettsia* Species	Study Site ^1^	No. of Individuals	Maximal Identity (%)	GenBank ID
ecotone	*Ixodes ricinus*	F	*Rickettsia helvetica*	S17	1	100	MF163037
					1	99.9	
				S16	1	100	
				S7	1	100	
				S8	1	99.9	
				S12	1	100	
				S9	1	100	
				S5	1	100	
		M		S16	1	100	
				S9	1	100	
					1	100	
				S5	1	100	
		N		S10	1	100	
				S9	1	99.9	
					1	100	
					1	99.75	
					1	100	
					1	100	
				S7	1	99.9	
					1	100	
				S5	2	100	
	*Dermacentor reticulatus*	F	*Rickettsia raoultii*	S2	1	99.9	KU723537
					5	99.9	MG811717
					3	100	
		M			1	99.9	MF002526
forest	*I. ricinus*	F	*R. helvetica*	S9	1	99.8	MF163037
					1	100	
				S8	1	100	KP866151
				S3	1	99.5	MF163037
				S14	1	100	
		M		S9	1	99.8	
					2	100	
				S3	1	100	
		N		S14	1	100	
					1	98	
				S12	1	99.9	
					1	100	
				S10	1	99.8	
				S9	3	99.8	
					15	100	
				S7	1	100	
				S8	3	100	
				S5	2	100	
				S3	1	99.7	
					9	100	
					1	99.9	
				S15	1	100	
				S13	1	100	
				S6	1	100	
	*D. reticulatus*	F	*R. raoultii*	S2	1	100	MF002526
		M		S3	1	99.9	MG811717
forest	*Apodemus flavicollis*	na	*R. helvetica*	S15	1	100	MF163037
				S13	1	100	

No.: Number; ID: Identification; F: Female; M: Male; N: Nymph; and na: not available. ^1^ Map of study sites with *Rickettsia* positive samples is available in Appendix (Figure A1).

## Data Availability

The dataset generated and analysed during the current study is available from the corresponding author.

## Appendix A

**Table A1:** Flagged tick individuals per species, per season, and in total, including larvae, nymphs, and adults.

	Absolute Number of Ticks (Percentage in Each Season)			
Tick Species	Spring	Summer	Autumn	Total
*Ixodes ricinus* complex ^1^	789 (77.2%)	203 (19.9%)	30 (2.9%)	1022 (100%)
*Dermacentor reticulatus*	37 (39.8%)	1 (1.1%)	55 (59.1%)	93 (100%)
**Total**	**826 (74.1%)**	**204 (18.3%)**	**85 (7.6%)**	**1115 (100%)**

^1^ *Ixodes ricinus* and *Ixodes inopinatus*.

**Table A2:** Results of a generalised linear mixed model for effects of tick species, life stage, habitat, and season on *Rickettsia* spp. infection probability in tick samples in total and in *Ixodes ricinus* complex ticks.

Factor	Estimate	Standard Error	Z Value	Probability (>|z|)
Total				
Intercept	−2.64	0.66	−4.03	**5.67** **×** **10****^−05^ *****
*D. reticulatus* vs. *I. ricinus* complex	−3.96	0.64	−6.17	**6.69** **×** **10****^−10^ *****
Adult vs. Nymph	−0.63	0.27	−2.33	0.02 *
Ecotone vs. Forest	−0.10	0.26	−0.37	0.71
Autumn vs. Spring	4.75	0.80	5.93	**3.05** **×** **10****^−09^ *****
Autumn vs. Summer	4.79	0.86	5.57	**2.57** **×** **10****^−08^ *****
*Ixodes ricinus*				
Intercept	−1.74	0.79	−2.19	**0.03 ***
Adult vs. Nymph	−0.79	0.27	−2.79	**0.01 ****
Ecotone vs. Forest	−0.15	0.28	−0.53	0.59
Autumn vs. Spring	−0.18	0.77	−0.24	0.81
Autumn vs. Summer	0.23	0.78	0.29	0.77

Significance codes: ***—<0.001; **—0.001; *—0.01.

**Table A3:** Results of a generalised linear mixed model with binomial error distribution for effects of habitat, season, sex, and small mammal species on *Rickettsia* spp. infection probability in small mammals in total and in the three most abundant small mammal species (*Apodemus flavicollis*, *Clethrionomys glareolus*, and *Microtus arvalis*). NA: not available.

Factor	Estimate	Standard Error	Z Value	Probability (>|z|)
**Total**				
Intercept	−4.09	0.66	−6.20	**5.78** **×** **10****^−10^ *****
Grassland vs. Forest	1.83	0.40	4.56	**5.22** **×** **10****^−06^ *****
Spring vs. summer	0.55	0.34	1.62	0.11
*A. agrarius* vs. *A. flavicollis*	0.00	0.46	0.01	1.00
*A. agrarius* vs. *A. sylvaticus*	0.70	0.50	1.38	0.17
*A. agrarius* vs. *M. arvalis*	0.44	0.53	0.83	0.41
*A. agrarius* vs. *Cl. glareolus*	−1.046	0.49	−2.12	**0.03 ***
***Apodemus flavicollis***				
Intercept	−17.73	223.46	−0.08	0.94
Female vs. Male	−0.10	0.63	−0.17	0.87
Grassland vs. Forest	16.58	223.46	0.07	0.94
Spring vs. Summer	−0.37	0.58	−0.64	0.52
***Clethrionomys glareolus***				
Intercept	−1.32	1.70	−0.78	0.44
Female vs. Male	−0.42	1.00	−0.42	0.67
Grassland vs. Forest	−0.66	1.42	−0.47	0.64
Spring vs. Summer	−0.17	0.75	−0.23	0.82
***Microtus arvalis***				
Intercept	−5.20	1.30	−3.98	**6.8** **×** **10****^−05^ *****
Female vs. Male	1.06	0.86	1.23	0.22
Grassland vs. Forest	1.97	0.60	3.31	**0.000948** ***
Spring vs. Summer	1.28	1.09	1.18	0.24

Significance codes: ***—<0.001; *—0.01

**Table A4:** Co-infections of *Borrelia* spp. and *Rickettsia* spp. in *I. ricinus* ticks. Data of *Borrelia* spp. investigation were available from Król et al. [32]. No.: Number; F: female; M: male; and N: nymph.

Species No. of Co-Infected Ticks/Total (%)	Year No. of Co-Infected Ticks/Total (%)	Season No. of Co-Infected Ticks/Total (%)	Habitat No. of Co-Infected Ticks/Total (%)	*Borrelia* spp.	*Rickettsia* spp.
*I. ricinus* complex 9/1094 (0.8)	2018 5/565 (0.9)	spring 5/436 (1.1)	ecotone 3/201 (1.5)	*B. valaisiana*	*R. helvetica*
				*B. afzelii*	
				*Borrelia* spp.	
			forest 2/235 (0.9)	*B. valaisiana*	
				*B. afzelii*	
	2019 4/529 (0.8)	spring 3/386 (0.8)	ecotone 1/101 (1.0)	*Borrelia* spp.	
			forest 2/285 (0.7)		
		summer 1/141 (0.7)	forest 1/22 (4.5)		

**Table A5:** Co-infections of *Borrelia* spp. and *Rickettsia* spp. in small mammals. Data of *Borrelia* spp. investigation were available from Król et al. [32]. No.: Number.

No. of Co-Infected Animals/Total (%)	Year No. of Co-Infected Animals/Total (%)	Season No. of Co-Infected Animals/Total (%)	Habitat No. of Co-Infected Animals/Total (%)	Species No. of Co-Infected Animals/Total (%)	*Borrelia* spp.	*Rickettsia* spp.
9/1167 (0.8)	2017 1/290 (0.3)	Summer 1/290 (0.3)	Forest 1/152 (0.7)	*A. flavicollis* 1/72 (1.4)	*Borrelia* spp.	*Rickettsia* spp.
	2018 6/94 (6.4)	Spring 1/23 (4.3)	Grassland 1/12 (8.3)	*M. arvalis* 1/12 (8.3)		
		Summer 5/71 (7.0)	Grassland 1/40 (2.5)	*M. arvalis* 1/33 (3.0)		
			Forest 4/31 (12.9)	*S. araneus* 2/7 (28.6)		
				*Cl. glareolus* 2/12 (16.7)		
	2019 2/783 (0.3)	Summer 2/638 (0.3)	Forest 2/372 (0.5)	*Cl. glareolus* 1/172 (0.6)		
				*M. arvalis* 1/19 (5.3)		

**Table A6:** Results from the co-occurrence seasonal analysis for all trapped ticks and mammals. Alpha maximum likelihood estimator (MLE) can be interpreted as an odds ratio for co-occurrence. The accompanying confidence interval (CI) was calculated according to Blaker [80].

Class	Season	Alpha MLE	Blaker CI (Lower)	Blaker CI (Upper)	*p*-Value
Arachnida (ticks)	Spring	−0.72	−1.60	0.00	0.06
	Summer	−0.54	−3.62	1.39	1.00
	Autumn	ND ^1^	ND ^1^	ND ^1^	ND ^1^
Mammalia (Small mammals)	Spring	0.09	−3.05	2.05	1.00
	Summer	0.32	−0.55	1.11	0.51

^1^ ND: not determined; no determination possible due to low numbers of ticks in autumn.

## References

[1] Petney T.N., Pfäffle M.P., Skuballa J.D. (2012). An annotated checklist of the ticks (Acari: Ixodida) of Germany. Syst. Appl. Acarol..

[2] Gray J., Kahl O., Zintl A. (2021). What do we still need to know about *Ixodes ricinus*?. Ticks Tick-Borne Dis..

[3] Stanko M., Derdáková M., Špitalská E., Kazimírová M. (2021). Ticks and their epidemiological role in Slovakia: From the past till present. Biologia.

[4] Medlock J.M., Hansford K.M., Bormane A., Derdakova M., Estrada-Peña A., George J.-C., Golovljova I., Jaenson T.G.T., Jensen J.-K., Jensen P.M. (2013). Driving forces for changes in geographical distribution of *Ixodes ricinus* ticks in Europe. Parasites Vectors.

[5] Drehmann M., Springer A., Lindau A., Fachet K., Mai S., Thoma D., Schneider C.R., Chitimia-Dobler L., Bröker M., Dobler G. (2020). The Spatial Distribution of Dermacentor Ticks (Ixodidae) in Germany—Evidence of a Continuing Spread of Dermacentor reticulatus. Front. Veter-Sci..

[6] Burri C., Schumann O., Schumann C., Gern L. (2014). Are *Apodemus* spp. mice and *Myodes glareolus* reservoirs for *Borrelia miyamotoi*, *Candidatus* Neoehrlichia mikurensis, *Rickettsia helvetica*, *R. monacensis* and *Anaplasma phagocytophilum*?. Ticks Tick-Borne Dis..

[7] Kraft R. (2008). Mäuse und Spitzmäuse in Bayern Verbreitung, Lebensraum, Bestandssituation.

[8] Krawczyk A.I., van Duijvendijk G.L.A., Swart A., Heylen D., Jaarsma R.I., Jacobs F.H.H., Fonville M., Sprong H., Takken W. (2020). Effect of rodent density on tick and tick-borne pathogen populations: Consequences for infectious disease risk. Parasites Vectors.

[9] Parola P., Paddock C.D., Socolovschi C., Labruna M.B., Mediannikov O., Kernif T., Abdad M.Y., Stenos J., Bitam I., Fournier P.-E. (2013). Update on Tick-Borne Rickettsioses around the World: A Geographic Approach. Clin. Microbiol. Rev..

[10] Kim H.K. (2022). *Rickettsia*-Host-Tick Interactions: Knowledge Advances and Gaps. Infect. Immun..

[11] Merhej V., Raoult D. (2010). Rickettsial evolution in the light of comparative genomics. Biol. Rev. Camb. Philos. Soc..

[12] Hauck D., Jordan D., Springer A., Schunack B., Pachnicke S., Fingerle V., Strube C. (2020). Transovarial transmission of *Borrelia* spp., *Rickettsia* spp. and *Anaplasma phagocytophilum* in *Ixodes ricinus* under field conditions extrapolated from DNA detection in questing larvae. Parasites Vectors.

[13] Raoult D., Roux V. (1997). Rickettsioses as paradigms of new or emerging infectious diseases. Clin. Microbiol. Rev..

[14] Socolovschi C., Mediannikov O., Raoult D., Parola P. (2009). The relationship between spotted fever group *Rickettsiae* and Ixodid ticks. Veter-Res..

[15] Karbowiak G., Biernat B., Stańczak J., Szewczyk T., Werszko J. (2016). The role of particular tick developmental stages in the circulation of tick-borne pathogens affecting humans in Central Europe. 3. Rickettsiae. Ann. Parasitol..

[16] Sprong H., Wielinga P.R., Fonville M., Reusken C., Brandenburg A.H., Borgsteede F., Gaasenbeek C., van der Giessen J.W. (2009). *Ixodes ricinus* ticks are reservoir hosts for *Rickettsia helvetica* and potentially carry flea-borne *Rickettsia* species. Parasites Vectors.

[17] Galfsky D., Król N., Pfeffer M., Obiegala A. (2019). Long-term trends of tick-borne pathogens in regard to small mammal and tick populations from Saxony, Germany. Parasites Vectors.

[18] Wölfel R., Terzioglu R., Kiessling J., Wilhelm S., Essbauer S., Pfeffer M., Dobler G. (2006). *Rickettsia* spp. in *Ixodes ricinus* Ticks in Bavaria, Germany. Ann. N. Y. Acad. Sci..

[19] May K., Strube C. (2014). Prevalence of Rickettsiales (*Anaplasma phagocytophilum* and *Rickettsia* spp.) in hard ticks (*Ixodes ricinus*) in the city of Hamburg, Germany. Parasitol. Res..

[20] Obiegala A., Oltersdorf C., Silaghi C., Kiefer D., Kiefer M., Woll D., Pfeffer M. (2016). *Rickettsia* spp. in small mammals and their parasitizing ectoparasites from Saxony, Germany. Veter-Parasitol. Reg. Stud. Rep..

[21] Tappe J., Strube C. (2013). *Anaplasma phagocytophilum* and *Rickettsia* spp. infections in hard ticks (*Ixodes ricinus*) in the city of Hanover (Germany): Revisited. Ticks Tick-Borne Dis..

[22] Azagi T., Hoornstra D., Kremer K., Hovius J.W.R., Sprong H. (2020). Evaluation of Disease Causality of Rare *Ixodes ricinus*-Borne Infections in Europe. Pathogens.

[23] de Sousa R., dos Santos M.L., Cruz C., Almeida V., Garrote A.R., Ramirez F., Seixas D., Manata M.J., Maltez F. (2022). Rare Case of Rickettsiosis Caused by *Rickettsia monacensis*, Portugal, 2021. Emerg. Infect. Dis..

[24] Buczek W., Koman-Iżko A., Buczek A., Bartosik K., Kulina D., Ciura D. (2020). Spotted fever group rickettsiae transmitted by Dermacentor ticks and determinants of their spread in Europe. Ann. Agric. Environ. Med..

[25] Parola P., Rovery C., Rolain J.M., Brouqui P., Davoust B., Raoult D. (2009). *Rickettsia slovaca* and *R. raoultii* in Tick-borne Rickettsioses. Emerg. Infect. Dis..

[26] Rieg S., Schmoldt S., Theilacker C., de With K., Wölfel S., Kern W.V., Dobler G. (2011). Tick-borne lymphadenopathy (TIBOLA) acquired in Southwestern Germany. BMC Infect. Dis..

[27] Špitalská E., Štefanidesová K., Kocianová E., Boldiš V. (2012). *Rickettsia slovaca* and *Rickettsia raoultii* in *Dermacentor marginatus* and *Dermacentor reticulatus* ticks from Slovak Republic. Exp. Appl. Acarol..

[28] Rizzoli A., Hauffe H.C., Carpi G., Vourc’h G.I., Neteler M., Rosà R. (2011). Lyme borreliosis in Europe. Eurosurveillance.

[29] Reye A.L., Hübschen J.M., Sausy A., Muller C.P. (2010). Prevalence and Seasonality of Tick-Borne Pathogens in Questing *Ixodes ricinus* Ticks from Luxembourg. Appl. Environ. Microbiol..

[30] Špitalská E., Boldiš V., Derdáková M., Selyemová D., Tarageľová V.R. (2014). Rickettsial infection in *Ixodes ricinus* ticks in urban and natural habitats of Slovakia. Ticks Tick-Borne Dis..

[31] Knoll S., Springer A., Hauck D., Schunack B., Pachnicke S., Strube C. (2021). Regional, seasonal, biennial and landscape-associated distribution of *Anaplasma phagocytophilum* and *Rickettsia* spp. infections in *Ixodes* ticks in northern Germany and implications for risk assessment at larger spatial scales. Ticks Tick-Borne Dis..

[32] Król N., Obiegala A., Imholt C., Arz C., Schmidt E., Jeske K., Ulrich R.G., Rentería-Solís Z., Jacob J., Pfeffer M. (2022). Diversity of *Borrelia burgdorferi* sensu lato in ticks and small mammals from different habitats. Parasites Vectors.

[33] Estrada-Peña A., Mihalca A.D., Petney T.N. (2017). Ticks of Europe and North Africa: A guide to species identification.

[34] Siuda K. (1993). Kleszcze Polski (Acari: Ixodida): Systematyka i Rozmieszczenie.

[35] Estrada-Peña A., Nava S., Petney T. (2014). Description of all the stages of *Ixodes inopinatus* n. sp. (Acari: Ixodidae). Ticks Tick-Borne Dis..

[36] Jeske K., Weber S., Pfaff F., Imholt C., Jacob J., Beer M., Ulrich R.G., Hoffmann D. (2019). Molecular Detection and Characterization of the First Cowpox Virus Isolate Derived from a Bank Vole. Viruses.

[37] Jeske K., Jacob J., Drewes S., Pfeffer M., Heckel G., Ulrich R.G., Imholt C. (2021). Hantavirus–*Leptospira* coinfections in small mammals from central Germany. Epidemiol. Infect..

[38] Wölfel R., Essbauer S., Dobler G. (2008). Diagnostics of tick-borne rickettsioses in Germany: A modern concept for a neglected disease. Int. J. Med. Microbiol..

[39] Roux V., Raoult D. (2000). Phylogenetic analysis of members of the genus Rickettsia using the gene encoding the outer-membrane protein rOmpB (ompB). Int. J. Syst. Evol. Microbiol..

[40] Bates D., Mächler M., Bolker B., Walker S. (2015). Fitting Linear Mixed-Effects Models Using lme4. J. Stat. Softw..

[41] Mainali K.P., Slud E., Singer M.C., Fagan W.F. (2022). A better index for analysis of co-occurrence and similarity. Sci. Adv..

[42] Mainali K.P., Slud E., Mainali M.K. Package ‘CooccurrenceAffinity’. https://cran.r-project.org/web//packages/CooccurrenceAffinity/CooccurrenceAffinity.pdf.

[43] Speck S., Kern T., Aistleitner K., Dilcher M., Dobler G., Essbauer S. (2018). In vitro studies of *Rickettsia*-host cell interactions: Confocal laser scanning microscopy of *Rickettsia helvetica*-infected eukaryotic cell lines. PLoS Neglected Trop. Dis..

[44] Kartashov M.Y., Glushkova L.I., Mikryukova T.P., Korabelnikov I.V., Egorova Y.I., Tupota N.L., Protopopova E.V., Konovalova S.N., Ternovoi V.A., Loktev V.B. (2017). Detection of *Rickettsia helvetica* and *Candidatus* R. tarasevichiae DNA in *Ixodes persulcatus* ticks collected in Northeastern European Russia (Komi Republic). Ticks Tick-Borne Dis..

[45] Rubel F., Brugger K., Chitimia-Dobler L., Dautel H., Meyer-Kayser E., Kahl O. (2021). Atlas of ticks (Acari: Argasidae, Ixodidae) in Germany. Exp. Appl. Acarol..

[46] Schulz M., Mahling M., Pfister K. (2014). Abundance and seasonal activity of questing *Ixodes ricinus* ticks in their natural habitats in southern Germany in 2011. J. Vector Ecol..

[47] Černý J., Lynn G., Hrnková J., Golovchenko M., Rudenko N., Grubhoffer L. (2020). Management Options for *Ixodes ricinus*-Associated Pathogens: A Review of Prevention Strategies. Int. J. Environ. Res. Public Health.

[48] Nowak-Chmura M., Siuda K. (2012). Ticks of Poland. Review of contemporary issues and latest research. Ann. Parasitol..

[49] Kohn M., Krücken J., McKay-Demeler J., Pachnicke S., Krieger K., von Samson-Himmelstjerna G. (2018). Dermacentor reticulatus in Berlin/Brandenburg (Germany): Activity patterns and associated pathogens. Ticks Tick-Borne Dis..

[50] Von Blanckenhagen F., Städtler T. (2011). Small mammal communities in agricultural landscapes in Germany: Review of field data over the last decade. Julius-Kühn-Archiv.

[51] Hotopp I., Walther B., Fuelling O., Reil D., Hesse C., Below D.A., Imholt C., Jacob J. (2022). Habitat and Season Effects on Small Mammal Bycatch in Live Trapping. Biology.

[52] Boyard C., Vourc’h G., Barnouin J. (2008). The relationships between *Ixodes ricinus* and small mammal species at the woodland–pasture interface. Exp. Appl. Acarol..

[53] Hauer S., Ansorge H., Zöphel U. (2009). Atlas der Säugetiere Sachsens: Naturschutz und Landschaftspflege.

[54] Dobler G., Wölfel R. (2009). Typhus and Other Rickettsioses: Emerging infections in Germany. Dtsch Arztebl. Int..

[55] Nilsson K. (2009). Septicaemia with *Rickettsia helvetica* in a patient with acute febrile illness, rash and myasthenia. J. Infect..

[56] Hildebrand J., Perec-Matysiak A., Popiołek M., Merta D., Myśliwy I., Buńkowska-Gawlik K. (2022). A molecular survey of spotted fever group rickettsiae in introduced raccoons (*Procyon lotor*). Parasites Vectors.

[57] Mendoza-Roldan J.A., Manoj R.R.S., Latrofa M.S., Iatta R., Annoscia G., Lovreglio P., Stufano A., Dantas-Torres F., Davoust B., Laidoudi Y. (2021). Role of reptiles and associated arthropods in the epidemiology of rickettsioses: A one health paradigm. PLOS Neglected Trop. Dis..

[58] Fischer S., Spierling N.G., Heuser E., Kling C., Schmidt S., Rosenfeld U.M., Reil D., Imholt C., Jacob J., Ulrich R.G. (2018). High prevalence of *Rickettsia helvetica* in wild small mammal populations in Germany. Ticks Tick-Borne Dis..

[59] Mysterud A., Byrkjeland R., Qviller L., Viljugrein H. (2015). The generalist tick *Ixodes ricinus* and the specialist tick *Ixodes trianguliceps* on shrews and rodents in a northern forest ecosystem– a role of body size even among small hosts. Parasites Vectors.

[60] Mardosaitė-Busaitienė D., Radzijevskaja J., Balčiauskas L., Paulauskas A. (2018). First detection of *Rickettsia helvetica* in small mammals in Lithuania. New Microbes New Infect..

[61] Schex S., Dobler G., Riehm J., Müller J., Sormunen J.J., Penttinen R., Klemola T., Hänninen J., Vuorinen I., Laaksonen M. (2011). *Rickettsia* spp. in Wild Small Mammals in Lower Bavaria, South-Eastern Germany. Vector-Borne Zoonotic Dis..

[62] Obiegala A., Król N., Oltersdorf C., Nader J., Pfeffer M. (2017). The enzootic life-cycle of Borrelia burgdorferi (sensu lato) and tick-borne rickettsiae: An epidemiological study on wild-living small mammals and their ticks from Saxony, Germany. Parasites Vectors.

[63] Stańczak J., Racewicz M., Michalik J., Cieniuch S., Sikora B., Skoracki M. (2009). Prevalence of infection with *Rickettsia helvetica* in feeding ticks and their hosts in western Poland. Clin. Microbiol. Infect..

[64] Svoboda P., Dobler G., Markotić A., Kurolt I.-C., Speck S., Habuš J., Vucelja M., Krajinović L.C., Tadin A., Margaletić J. (2014). Survey for Hantaviruses, Tick-Borne Encephalitis Virus, and *Rickettsia* spp. in Small Rodents in Croatia. Vector-Borne Zoonotic Dis..

[65] Miťková K., Berthová L., Kalúz S., Kazimírová M., Burdová L., Kocianová E. (2015). First detections of *Rickettsia helvetica* and *R. monacensis* in ectoparasitic mites (Laelapidae and Trombiculidae) infesting rodents in south-western Slovakia. Parasitol. Res..

[66] Martello E., Mannelli A., Grego E., Ceballos L.A., Ragagli C., Stella M.C., Tomassone L. (2019). *Borrelia burgdorferi* sensu lato and spotted fever group rickettsiae in small rodents and attached ticks in the Northern Apennines, Italy. Ticks Tick-Borne Dis..

[67] Pluta S., Hartelt K., Oehme R., Mackenstedt U., Kimmig P. (2010). Prevalence of *Coxiella burnetii* and *Rickettsia* spp. in ticks and rodents in southern Germany. Ticks Tick-Borne Dis..

[68] Chang Q.-C., Hu Y., Wu T.-T., Ma X.-X., Jiang B.-G., Jia N., Wang A.-Q., Jiang J.-F. (2022). The Role of Ranged Horses in Eco-Epidemiology of *Rickettsia raoultii* Infection in China. Front. Microbiol..

[69] Liu G., Zhao S., Tan W., Hornok S., Yuan W., Mi L., Wang S., Liu Z., Zhang Y., Hazihan W. (2021). Rickettsiae in red fox (*Vulpes vulpes*), marbled polecat (*Vormela peregusna*) and their ticks in northwestern China. Parasites Vectors.

[70] Zeroual F., Leulmi H., Bitam I., Benakhla A. (2018). Molecular evidence of *Rickettsia slovaca* in spleen of wild boars in northeastern Algeria. New Microbes New Infect..

[71] Stańczak J., Biernat B., Racewicz M., Zalewska M., Matyjasek A. (2017). Prevalence of different *Rickettsia* spp. in *Ixodes ricinus* and *Dermacentor reticulatus* ticks (Acari: Ixodidae) in north-eastern Poland. Ticks Tick-Borne Dis..

[72] Schorn S., Pfister K., Reulen H., Mahling M., Silaghi C. (2011). Occurrence of *Babesia* spp., *Rickettsia* spp. and *Bartonella* spp. in *Ixodes ricinus* in Bavarian public parks, Germany. Parasites Vectors.

[73] Silaghi C., Gilles J., Höhle M., Pradel I., Just F.T., Fingerle V., Küchenhoff H., Pfister K. (2008). Prevalence of spotted fever group rickettsiae in *Ixodes ricinus* (Acari: Ixodidae) in southern Germany. J. Med. Entomol..

[74] Samoylenko I., Shpynov S., Raoult D., Rudakov N., Fournier P.-E. (2009). Evaluation of Dermacentor species naturally infected with *Rickettsia raoultii*. Clin. Microbiol. Infect..

[75] Hildebrandt A., Krämer A., Sachse S., Straube E. (2010). Detection of *Rickettsia* spp. and *Anaplasma phagocytophilum* in *Ixodes ricinus* ticks in a region of Middle Germany (Thuringia). Ticks Tick-Borne Dis..

[76] Blazejak K., Janecek E., Strube C. (2017). A 10-year surveillance of Rickettsiales (*Rickettsia* spp. and *Anaplasma phagocytophilum*) in the city of Hanover, Germany, reveals *Rickettsia* spp. as emerging pathogens in ticks. Parasites Vectors.

[77] Kantsø B., Svendsen C.B., Jensen P.M., Vennestrøm J., Krogfelt K.A. (2010). Seasonal and habitat variation in the prevalence of Rickettsia helvetica in *Ixodes ricinus* ticks from Denmark. Ticks Tick-Borne Dis..

[78] Raulf M.-K., Jordan D., Fingerle V., Strube C. (2018). Association of *Borrelia* and *Rickettsia* spp. and bacterial loads in *Ixodes ricinus* ticks. Ticks Tick-Borne Dis..

[79] Knoll S., Springer A., Hauck D., Schunack B., Pachnicke S., Fingerle V., Strube C. (2021). Distribution of *Borrelia burgdorferi* s.l. and *Borrelia miyamotoi* in *Ixodes* tick populations in Northern Germany, co-infections with Rickettsiales and assessment of potential influencing factors. Med. Veter-Èntomol..

[80] Blaker H. (2000). Confidence curves and improved exact confidence intervals for discrete distributions. Can. J. Stat..

